# Anti-thrombotic effects of α-linolenic acid isolated from *Zanthoxylum bungeanum Maxim* seeds

**DOI:** 10.1186/1472-6882-14-348

**Published:** 2014-09-23

**Authors:** Qian Yang, Weidong Cao, Xuanxuan Zhou, Wei Cao, Yanhua Xie, Siwang Wang

**Affiliations:** Department of Materia Medica, School of Pharmacology, Fourth Military Medical University, Xi’an, Shaanxi China; Department of Neurosurgery, Xijing Hospital, Fourth Military Medical University, Xi’an, Shaanxi China

**Keywords:** Alpha-linolenic acid, Anti-thrombotic, Zanthoxylum bungeanum Maxim seeds, Linoleic acid, Unsaturated fatty acids

## Abstract

**Background:**

The current study was to evaluate the anti-thrombotic effect of alpha-linolenic acid (ALA) which was isolated and purified from *Jiaomu in vivo*.

**Methods:**

The seeds were crushed and subsequently subjected to saponification, acid hydrolysis, gradient freezing, urea inclusion and complexation of silver nitrate to obtain the unsaturated fatty acids. The chemical characteristics of isolated ALA were validated by ^1^HNMR, ^13^CNMR and mass spectrometry, and then the anti-thrombotic effect of ALA and its mixture with linoleic acid (1:1) were evaluated in the following experiments.

**Results:**

The alpha-linolenic acid was isolated and purified from *Jiaomu* through our newly established methods. ALA and its mixture with linoleic acid can prolong the hemorrhage and coagulation time as well as enhanced the survival rate of mice subjected to collagen-adrenaline induced thrombosis. In addition, the thrombosis on A-V bypass and platelet aggregation of rats will be reduced after treated with ALA or its mixture, and the expression level of Akt and PI3K protein decreased 26% and 31%, respectively.

**Conclusions:**

We designed and optimized a very simple and high-yield procedure to isolate ALA and linoleic acid mixture from seeds of *Zanthoxylum bungeanum Maxim* and demonstrated that such mixture can obtain a good anti-thrombotic effect through the modulation of PI3K/Akt signaling.

## Background

Alpha-linolenic acid (ALA), which belongs to a group of fatty acids named omega-3 fatty acids, is an essential fatty acid. Seed oils are the richest sources of ALA, especially those of rapeseed, soybeans, walnuts, flaxseed, perilla, chia and hemp. ALA can be obtained from thylakoid membranes of the green leaves of broadleaf plants [1, 2]. Similar to other essential fatty acids, ALA is critical for human bodily function given its conversion into the longer chain fatty acids eicosapentaenoic acid (EPA) and docosahexaenoic acid (DHA). ALA and its metabolic products, has long been known to reduce the risk of detrimental disorders including cardiac arrhythmia, high cholesterolemia, hypertension, thrombosis, allergy and cancer [3, 4]. It was reported that ALA may possess the anti-arrhythmic properties after investigation of the association between arrhythmia and dietary ALA intake accessed *via* updated food-frequency questionnaires [5]. Intake of omega-3 fatty acids reduces risk of fatal coronary heart events, perhaps by stabilizing myocardium and reducing risk of fatal arrhythmias [6]. The wide variety of ALA-exerted biological actions may have relevance to their involvements in several physiological and pathological processes. ALA defect is closely related to the prevalence of hypertension, diabetes mellitus, coronary heart disease, schizophrenia, Alzheimer’s disease, atherosclerosis and cancer, thus depicting the importance of adequate intake of ALA in daily life [7–9].

In spite of its crucial role in human health, dietary insufficiency in ALA is a rather common medical problem worldwide. As such, *Food and Agriculture Organization of the United Nations and World Health Organization* issued a joint statement, urging the necessity of increasing intake of ALA. Its annual demand has been over two million kilogram. Given ALA synthesis is still beyond our capability, it is of great importance to seek the natural sources of ALA.

*Jiaomu* (Semen Zanthozyli bungeani) is the seeds of *Zanthoxylum bungeanum Maxim* [10]. In the 1990s, groups of Chinese scientists discovered that the content of ALA in *Jiaomu* is about 17 ~ 24% [11, 12], solidifying *Jiaomu* as a rich source of ALA. Being seeds of *Zanthoxylum bungeanum Maxim*, a traditional Chinese cuisine agent, 120 million kilogram of Jiaomu are discarded in China each year. As such, *Jiaomu* could be a rich source of ALA as long as the obstacles in its chemical production can be rectified. Up to date, highly efficient procedure is still lacked for the isolation and purification of ALA.

The aggregation and activation of platelet can affect the development of myocardial infarction, stroke and unstable angina, and thus, the anti-platelet agents can be induced the obviously antithrombotic effect and used to prevent various cardiovascular disorders. Furthermore, it has been demonstrated that GP *IIb/IIIa* receptor which can regulate the process of hemostasis in the body and be involved in the thrombus formation and platelet adhesion. The clinical application of platelet glycoprotein *IIb/IIIa* (GP *IIb/IIIa*) antagonists can prevent the fibrinogen-mediated aggregation of activated platelets [13–15]. But so far, the detail and further underlying mechanism on the modulation of anti-thrombotic process was still not clear after ALA treatment.

In the current study, firstly, we attempted to design and optimize the chemical strategy in the extraction of ALA from raw *Jiaomu* and evaluated its anti-thrombotic effect *in vivo*. In addition, the underlying mechanism of above process was also investigated to evaluate the future application of *Jiaomu* in health and dietary areas.

## Methods

### Materials

Authenticated, quality-certified raw *Jiaomu* were purchased from the *Material Company of Hancheng* (*Shan’xi Province, China*). Standard ALA compound was obtained from the *Sigma Chemical Company* (*St Louis, MO, USA*). It was dissolved in normal saline containing 0.5% Tween-80 prior to use. Aspirin enteric-coated tablet was purchased from *Bailu Pharm Co. Ltd.*, (*Xi’an, China*). Other analytical reagents were obtained from the *Chemical Agent Factory of Xi’an* (*Xi’an, China*).

All mice weighing at 18 ~ 22 g and rats weighing at 250 ~ 300 g were provided by the animal center of *Fourth Military Medical University*. Animals were housed two per cage in a room controlled for temperature, lighting and humidity. All experiments were performed according to the ethical guidelines on animal care and were approved by the institutional animal care committee of Fourth Military Medical University.

### Preparation of ALA

#### Acid hydrolyze saponification

The seeds were crushed and sifted out (40 ~ 60 mesh). The powder was decocted with 5% NaOH for one hour at 65°C and filtrated to obtain the aqua part. Above process was repeated twice to combine the resulted filtrate. The solution was treated with H_2_SO_4_ (v/v, 1:1) to acidify to pH 2.0. The oil layer was separated and washed with water to pH 7.0 to get the mixtures of fatty acid.

#### Gradient freezing

The mixtures of fatty acid mixed with 95% NaOH (v/v, 1:1) were placed at 0°C for 6 ~ 7 h before filtration. Then the solution was continuously frozen at -5°C for 6 ~ 8 h, filtrated and washed off residues with chilled 95% NaOH. The solvent was distilled-off to obtain the lipid portion.

#### Urea inclusion

The urea was dissolved into 95% NaOH, and then added into the frozen mixtures of the fatty acid (oil: urea = 1:1). After a sufficient mixing, the mixture was subsequently cooled at 3 ~ 5°C, -6°C and -12°C for 6 ~ 8 h, and then quickly filtrated. The residue was washed off with 95% NaOH to obtain the oil after removing the solvent. The latter was bathed in water for 3 times, extracted with petrol ether, shaken with dried MgSO_4_ for 5 ~ 10 min, and then filtrated. After contraction, the obtained extracts contained mostly unsaturated fatty acid (50% ALA and 50% linoleic acid).

#### Complexation of silver nitrate

The mixtures of fatty acids were added into urea, mixed with AgNO_3_ solution at low temperature, stirred and then delaminated. The AgNO_3_ layer was partitioned in petrol ether and repeated 2 ~ 3 times. The petrol ether layers were combined and concentrated to yield ALA.

### Chemical analysis

The content of yielded ALA was analyzed by high-performance liquid chromatography (HPLC; Waters, Milford) using a C18 column (4.6 × 250 mm; 5 μM) in 90:10 (v/v) acetonitrile/ 1% acetic acid solution delivered at 1 ml/min as the mobile phase. Elution of compounds was detected at 205 nm with 2996 UV detector. ^1^HNMR, ^13^CNMR and Mass spectrometry measurements were also performed to validate the produced compound, using commercial standard ALA compound as the control.

### Hemorrhage time and coagulation time determination

All mice were randomly divided into the treated group with different dosage of ALA, negative (saline) and positive (aspirin) control groups. ALA treatment groups were received either pure ALA or its mixture with linoleic acid (*ALA: linoleic acid = 1:1*) at 50, 100, or 250 mg/kg for 10 days. Following the last administration, platelet-rich plasma (PRP) was prepared and obtained from blood samples according to the previous methods [16, 17]. Brifely, the sample containing 3.8% sodium citrate were centrifuged at 100 × g for 10 min at room temperature to obtain the PRP in supernatant. After that, the hemorrhage and coagulation time were determined by the related Kit (*PT and APTT Kit*) and Analyzer (*LG-PABER, China*).

### Thrombus formation analysis

Mice were grouped and treated as described above. After the last treatment, a mixture of collagen (3.57 mg/kg) and adrenaline (0.143 mg/kg) were injected into vena caudal vein of mouse to stimulate thrombus formation for 5 min through the effect of platelet activation [18]. Mortality was calculated to determine the survival rate.

### Artery-venous (A-V) bypass thrombosis and Platelets aggregation analysis

Rats were randomly divided into the treated and control groups according to the above procedure. Pure ALA compound or its mixture with linoleic acid (*ALA: linoleic acid = 1:1*) were administered at 35, 70 or 150 mg/kg for 10 days. A-V bypass thrombosis analysis was performed as previously described to determine the wet weight of thrombus [19].

Venous blood was collected by atraumatic venepuncture into vacuette tubes containing sodium citrate (3.8%, v/v) after anesthetized animals. Platelet-rich plasma (PRP) was prepared according to the above method using collagen I (22.0 μg/ml) as an inducer. Maximum aggregation rate and aggregation inhibition rate were determined [20].

### Effect of ALA on P-selectin and GP IIb/IIIa expression on washed platelets

Platelets were prepared as described above. P-selectin (*CD62*) and GP *IIb/IIIa* expression was measured using FITC-labeled antibody. Briefly [21], platelets were washed and centrifuged, and then blocked with 5% BSA solution. The antibody of P-selectin (*CD62*) and GP *IIb/IIIa* were incubated with sample for 30 min at 4°C in the dark, respectively. Flow cytometry (*FCM, Becton Dickinson, San Jose, CA, USA*) was performed to detect the expression of P-selectin and GP *IIb/IIIa*.

### Western bolt analysis

Western blotting procedure was performed to investigate the changes on related protein expression in the presence or absence of ALA. Briefly, platelets were prepared as described above. Protein was collected in the following buffer including 0.1 M NaCl, 0.01 M Tris–HCl (pH 7.6), 1% (w/v) Triton X-100, 1 mM EDTA (pH 8.0), 100 mg/ml PMSF, 1% (w/v) NP-40, and 1 μg/ml leupeptin. After centrifugation at 10,000 × *g* for 30 min at 4°C, the total protein was harvested and quantified by the BCA protein assay kit (*Pierce, Rockford, IL, USA*). After that, the protein was separated by SDS-PAGE and electrophoretically transferred onto a PVDF membrane according to the standard western blotting protocol. The membrane was incubated with a primary antibody against PI3K and Akt (1:1000; *Cell Signaling Technology, Beverly, MA, USA*) overnight at 4°C after blocking with 5% milk in PBST. The enhanced chemiluminescence system was used to detect the positive belt. The blot was also re-probed and corrected by staining with β-actin antibody (*Sigma, St Louis, MO, USA*).

### Statistical analysis

Data are presented as means ± SD. Differences between groups were evaluated by the student’s t test. *ANOVA* was applied to examine the difference in survival rate. All analyses were performed utilizing the SPSS software package (*Version 11.5, SPSS Inc., Chicago, IL*). Differences were considered statistically significant at p < 0.05.

## Results

### Chemical characteristics

**Figure 1:**
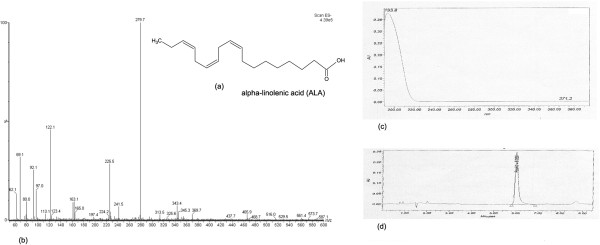
**Chemical characteristics of alpha-linolenic acid. (a)** Chemical structure of alpha-linolenic acid; **(b)** Negative ion mass spectra of alpha-linolenic acid, *m/z = 279.6;* **(c)** UV; **(d)** HPLC.

**Figure 2:**
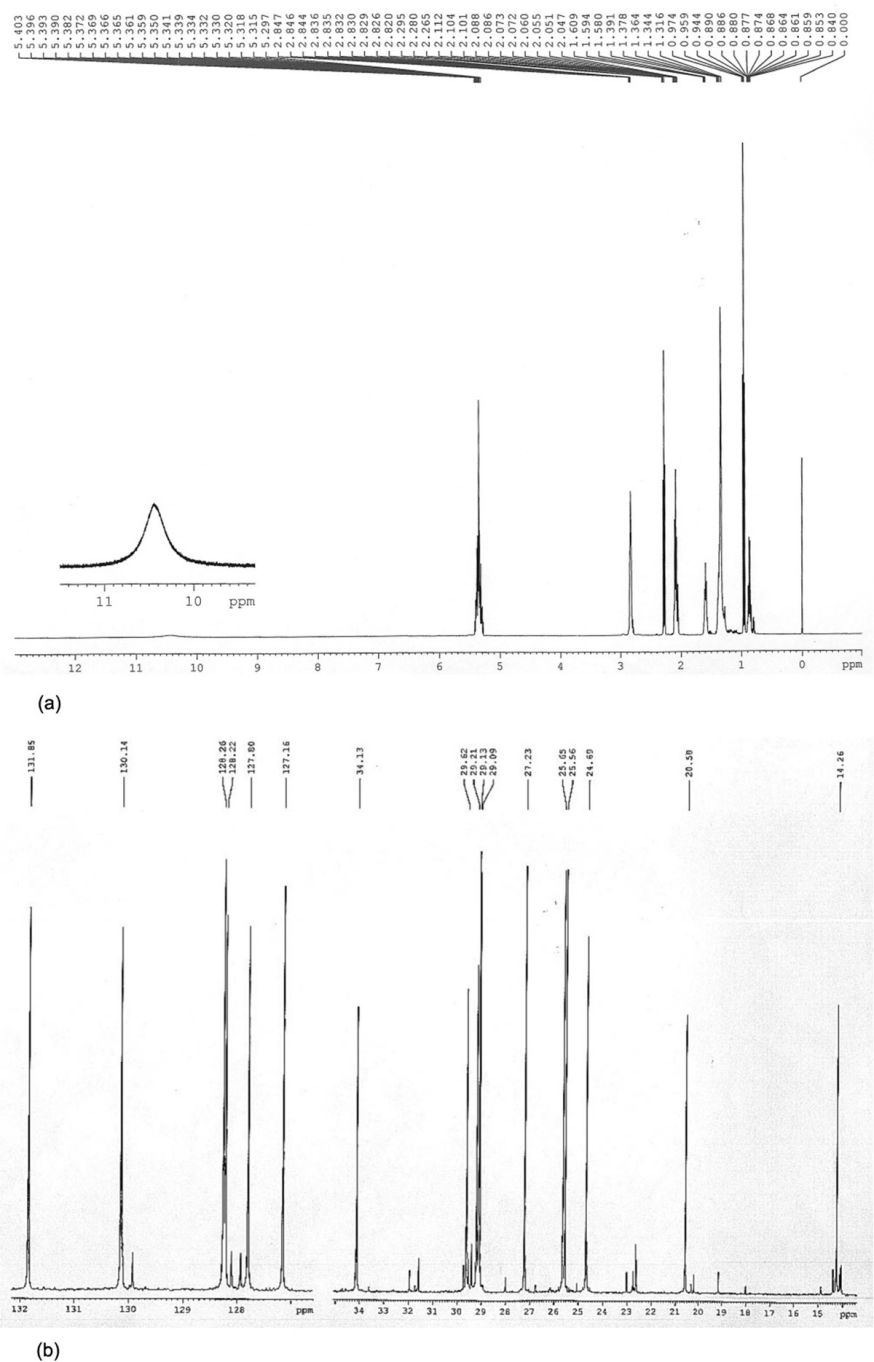
**NMR analysis data of alpha-linolenic acid. (a)** ^1^HNMR δ/ppm; **(b)** ^13^CNMR δ/ppm.

The characteristics of isolated and purified alpha-linolenic acid (ALA) from *Jiaomu* were evaluated through the following experiments. A Quattro Premier MS system (*Waters Corp., Milford, MA, USA*) operating under Masslynx 4.1 software was performed using liquid chromatography/electrospray ionization tandem mass spectrometry equipped with an electrospray ionization interface used to generate positive ions [M^-^H^+^] for the determination of ALA. The positive ion mass spectra of ALA showed stable molecular ions at *m/z = 279.6* (Figure 1b). The source temperature was 110°C. The electrospray capillary voltage was 3.0 kV, nitrogen was the desolvation gas (500 mL/min flow rate). Compounds were separated on a reversed-phase column by a Waters 2695 HPLC system. The column temperature was 25°C. The retention time of isolated ALA sample was 6.036 min, identical to that of the standard compound (Figure 1d). The purity of ALA ranged from 91.6% to 95.2%. NMR analysis data were presented as follows: ^*13*^*CNMR δ/ppm* (Figure 2a): 180.43 (C_1_), 134.13 (C_2_), 131.85 (C_9_), 130.14 (C_16_), 128.26 (C_12_), 128.22 (C_13_), 127.80 (C_15_), 127.16 (C_10_), 29.62 (C_6_), 29.21 (C_7_), 29.13 (C_5_), 29.09 (C_4_), 27.23 (C_8_), 25.65 (C_14_), 25.56 (C_11_), 24.69 (C_3_), 20.56 (C_17_), 14.26 (C_18_); ^*1*^*HNMR δ/ppm* (Figure 2b): 11.4 (br, s, 1H, -COOH), 5.3 ~ 5.4 (m, 6H, -CH-CH-, C_9, 10, 12, 13, 15, 16_), 2.8 (t, 4H, CH_2_, CH_2_, C_11, 14_), 2.35 (t, 2H, CH_2_, C_2_), 2.06 (m, 4H, CH_2_, C8, 17), 1.63 (m, 2H, CH_2_, C_3_), 1.32 (m, 8H, CH_2_, C_4, 5, 6, 7_), 0.98 (t, 3H, CH_3_, C_18_). And above data also provided the certainly evidence for efficiently isolated and purified methods of ALA from *Jiaomu*.

#### Effects of ALA on hemorrhage and coagulation time

**Figure 3:**
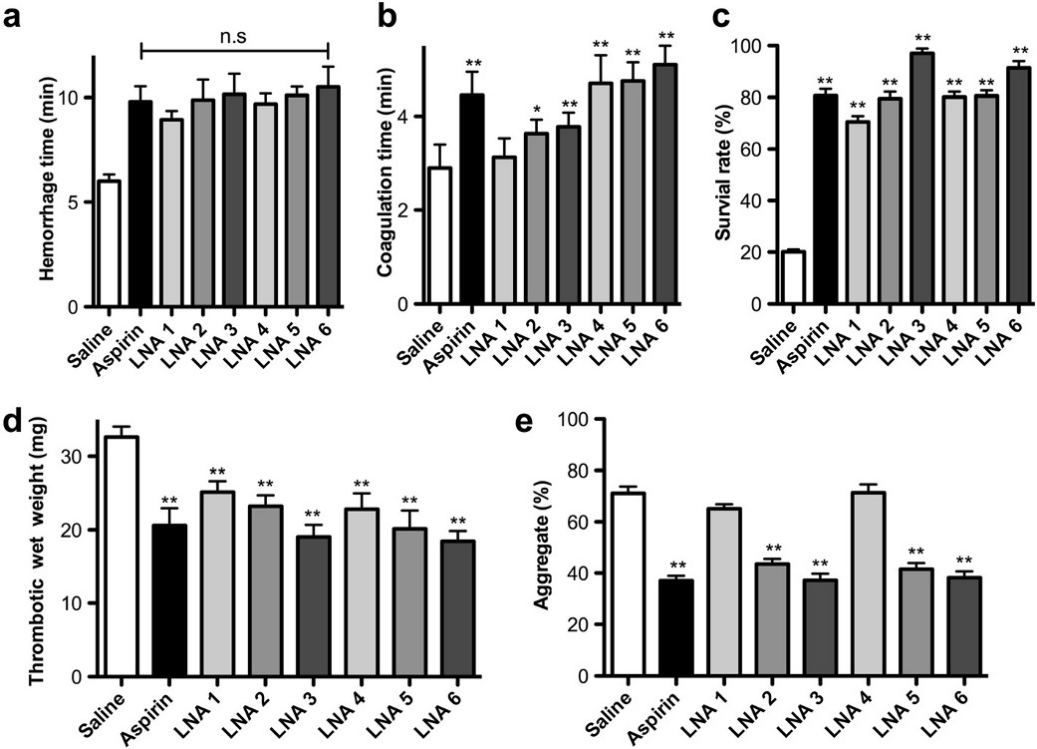
**Anti-thrombotic effects of alpha-linolenic acid. (a)** Effects of ALA on hemorrhage time; **(b)** Effects of ALA on coagulation time; **(c)** Effect of ALA on trombus formation analysis, mortality was calculated to determine the survival rate; **(d)** Effect of ALA on artery-venous (A-V) bypass thrombosis, the wet weight of thrombus was determined by subtracting the weight of dry cotton thread; **(e)** Rat platelets were isolated and platelet aggregation was determined *in vitro*. Aspirin (100 mg/kg), α-LNA 1–3 (pure ALA: 50, 100 and 250 mg/kg), α-LNA 4–6 (ALA mixture: 50, 100 and 250 mg/kg), * p < 0.05, ** p < 0.01 vs. control group.

To explore the effect of ALA on the stanching and coagulation functions of mice, we examined the hemorrhage and coagulation time of ALA or its mixture treated mice. As shown in Figure 3a or b, pure ALA with lower, middle or higher dosage (50, 100 or 250 mg/kg) prolonged the hemorrhage and coagulation time, respectively. The mixture composed of ALA and linoleic acid (1:1) also remarkably increased hemorrhage and coagulation time. For above positive effects on hemorrhage time, there is no clear difference among each treated groups, but for coagulation time, the effects displayed with a concentration-dependent manner both in pure and mixture treated groups. In the group of mixture with higher dosage, the coagulation time increased at least 35% compared with the saline treated group. Our results indicated that both ALA and the mixture treatment significantly inhibited blood standing and coagulation in mice.

#### Effects of ALA on thrombosis

We further determined the therapeutic effect of ALA and its mixture on stimulate thrombosis formation. Collagen and adrenaline efficiently induced thrombus formation in mice, as revealed by rapid mortality of most animals following the inducer treatment. Similar to aspirin, ALA enhanced the survival rate of the animals significantly (Figure 3c). After treated with 250 mg/kg ALA, the survival rate increased at least 3.5 times compared with saline treated group, indicating that ALA can completely inhibit collagen- and adrenaline-induced thrombosis in mice at this concentration. And for the mixture treated group with higher dosage, the similar effects were also observed and no difference between these two groups.

After treated with ALA or its mixture in rats, A-V bypass thrombosis was also evaluated in each group. Likewise, ALA and its mixture were found to inhibit collagen stimulated A-V bypass thrombosis in rats. Both pure ALA and mixture with lower, middle or high dosage (35, 70 or 175 mg/kg) suppressed A-V thrombus formation, respectively. Compared with saline treated group, the thrombotic wet weight decreased 33% and 42% after treated with ALA or its mixture group, respectively (Figure 3d). Our above results indicated that the ALA mixture offered a better therapeutic effect than the pure ALA compound.

#### Effects of ALA on collagen-induced platelet aggregation in rats

Moreover, we explored the effect of ALA and its mixture on the collagen-induced platelet aggregation (Figure 3e). Firstly, the effect of aspirin (100 mg/kg) on the inhibition of collagen stimulated platelet aggregation was observed *in vitro.* After treated with middle or high dosage (70 or 175 mg/kg) pure ALA, such inhibition was also occurred obviously, the platelet aggregation decreased 29% or 34% in middle or higher dosage group, respectively. But for lower dosage (35 mg/kg) group, there was no significant changes compared with saline treated group. Similar results were also observed after treated with ALA mixture, the platelet aggregation decreased 39% or 46% in middle or high dosage group, respectively.

#### Effect of ALA on P-selectin and GP IIb/IIIa expression

**Figure 4:**
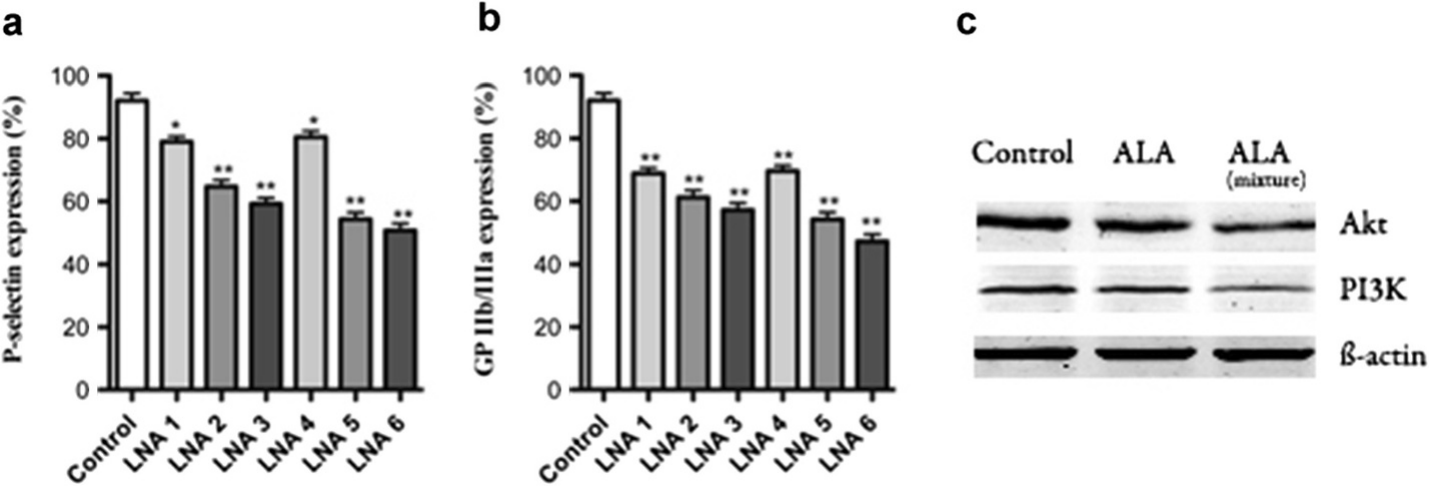
**Effect of ALA on P-selectin, GP IIb/IIIa expression and PI3K signaling.** Effect of ALA on P-selectin **(a)** and GP *IIb/IIIa* **(b)** expression (* p < 0.05, ** p < 0.01 vs. control group.); **(c)** western blot results showed the effect of ALA on PI3K and AKT expression. α-LNA 1–3 (pure ALA: 50, 100 and 250 mg/kg), α-LNA 4–6 (ALA mixture: 50, 100 and 250 mg/kg).

The effects on platelet P-selectin and GP *IIb/IIIa* expression were examined by FCM after treated with pure ALA or its mixture. As shown in Figure 4a, ALA reduced P-selectin secretion at 12%, 27% or 32% in the different group with lower, middle or higher dosage, respectively. And ALA mixture can reduce P-selectin secretion at 13%, 36% or 41%, respectively. In addition, the expression of GP *IIb/IIIa* decreased 25%, 31% or 35% in ALA treated group with lower, middle or higher dosage, respectively. Similar tendency on GP*IIb-IIIa* expression were observed after ALA mixture treatment, it decreased 24%, 38% or 45%, respectively (Figure 4b).

#### Effect of ALA on PI3K signaling

The expression level of Akt and PI3K, which are directly related to changes in thrombosis formation process, were examined after ALA and its mixture treatment through western blot analysis. The results showed that Akt expression decreased 26% in ALA treated group, and 35% in ALA mixture treated group, respectively. After treated with ALA, the inhibition on Akt changes was clearly. Furthermore, the expression of PI3K were also decreased 31% and 39% after treated with ALA and its mixture, respectively (Figure 4). Above results provided the direct evidence that both ALA and its mixture have the obvious anti-thrombosis effects which were related to the changes of PI3K or Akt protein expression level, and such effects of ALA on the protein changes was similar with ALA mixture treated group, which was the possible reason for the above similar tendency on anti-thrombosis effects between ALA and its mixture.

## Discussion

In the current study, our data provided the following evidences: (1) the highly purified ALA was isolated from *Jiaomu* through our newly established methods; (2) ALA and its mixture can reduce the thrombosis on A-V bypass and platelet aggregation; (3) The hemorrhage and coagulation time can be prolonged after treated with ALA and its mixture with linoleic acid; (4) ALA and its mixture application can reduce the P-selectin secretion and GP*IIb-IIIa* expression; (5) Anti-thrombotic effect of ALA was related to the expression of PI3K and Akt, which may be the possible mechanism of above process. And thus, above data demonstrate that ALA can affect the process of thrombotic through the modulation of PI3K/Akt signaling.

We designed and optimized the isolation procedure of ALA from *Jiaomu*, seeds of Z*anthoxylum bungeanum Maxim*, and demonstrated an important role of these seeds as a potential source of ALA. Given that most fatty acids exist along with lipid, we attempted to extract the fatty acid mixture by acid leaching following decoction in 5% NaOH. Our current approach was revealed to be more efficient than the traditional method in separating lipids from raw materials. Moreover, our method is more feasible for industrial production. Based on the various freezing points of fatty acids, we applied the gradient freezing method to facilitate removal of wax, saturated fatty acids, mono- and di-unsaturated fatty acids which should help to reduce consumption of urea and enhance its efficiency. As previously reported [22], urea can form stable inclusion with saturated and mono-unsaturated fatty acids at low temperature, which results in isolate poly-unsaturated fatty acids. Since urea also form inclusion with some of unsaturated fatty acids, it is crucial to maintain proper urea content in order to prevent the loss of ALA.

It was demonstrated that the function of blood stanching and coagulation can be represented through the evaluation on hemorrhage and coagulation time. According to our results, ALA treatment prolonged both indices in mice, consistent with the results from previously report [23]. Furthermore, ALA isolated from the seeds can greatly enhance the survival rate of mice suffering from collagen/adrenaline-induced thrombosis and decrease the mass of thrombus formed during A-V bypass. All these data collectively confirmed the *in vivo* activity of extracted ALA and thus demonstrated that the isolation procedure reported in our study not only increases the yield but also successfully maintains the biological activity of the compound of interest.

It has been evidenced that the process of platelet aggregation and thrombus formation was related to the P-selectin secretion and GP *IIb/IIIa* activation which was also cross-linked Akt activation [24, 25]. Our current results provided the certainly evidence that ALA can reduce P-selectin secretion and GP *IIb/IIIa* expression obviously. Furthermore, the platelet function, including activation, adhesion, spreading, and aggregation, was related to the changes on the signaling pathway of PI3K and Akt which was the main target of PI3K [26, 27]. Our results also showed that ALA reduced the expression level of PI3K and Akt. And such results demonstrated that the anti-thrombotic effect of ALA will be related to the PI3K signaling pathway.

In our current study, it was interested to find that the biological activity of ALA and linoleic acid (1:1) mixture is superior to that of the pure ALA. We speculated that it may be ascribed to the competition of linoleic acid with ALA in metabolism. Although the two acids have their specific metabolic pathways, they share the similar enzymes. ALA is metabolized into EPA and DHA by the action of a serious of desaturase and elongase, while linoleic acid is converted to arachidonic acid (AA). The competition between ALA and linoleic acid suppresses the production of AA, which competes with EPA for cyclooxygenase and lipooxidase and consequently inhibits the generation of leukotrienes and prostaglandins. According to our results, ALA and linoleic acid mixture at 1:1 can achieve a better anti-thrombosis efficiency than ALA alone, suggested that their relative ratio is crucial for the ultimate therapeutic effect. Notably, the urea inclusion method applied in the current study yielded the mixture, consisting of 50% ALA and 50% linoleic acid. The extracts from *Jiaomu* through urea inclusion cannot only achieve favorable therapeutic effect, but also greatly simplify the production procedure and costs.

## Conclusions

In summary, we designed and optimized a very simple and high-yield procedure to isolate ALA and linoleic acid mixture from seeds of *Zanthoxylum bungeanum Maxim*. The data demonstrated that such mixture can achieve a better anti-thrombosis effect than pure ALA through the modulation of PI3K/Akt signaling. Our results favor the seeds as a rich source of ALA and consolidate its future application in dietary and medical areas.
